# Meteorology and Health

**Published:** 1849-05

**Authors:** 


*Meteorology and Health.*—The Report of the New Orleans Board of Health, in the March number of the Medical Journal of that city, contains the following meteorological statistics, the bearing of which upon health will be perceived.

From observations made at different points in the valley of the Mississippi during a period of more than 20 years, the average quantity of rain that annually fell, amounted to about 2·5 feet prior to 1845; however, there has been a steady and rapid increase since over that average. That year (1845) the quantity of rain as ascertained by Mr. Lillie's rain guage, which is graduated to the thousandth part of an inch, was 2·419 feet, and there were 108 rainy days.

In 1846, the quantity of rain during the year was 6·39 feet, and there were 114 rainy days. In 1847, it had increased to 8·407 feet, which fell in 104 days.—

And in 1848, it amounted to the unusual quantity of 10·750 feet, and the number of rainy days was 116, as follows:—

| | | | | | | | |
|---|---|---|---|---|---|---|---|
| In January, | 1848, | 8 | rainy days—fall of rain | | 18·387 | inches. |
| In February, | " | 9 | do | do | 11·890 | " |
| In March | " | 6 | do | do | 2·705 | " |
| In April, | " | 8 | do | do | 7·643 | " |
| In May, | " | 5 | do | do | 11·675 | " |
| In June, | " | 16 | do | do | 30·306 | " |
| In July, | " | 22 | do | do | 14·110 | " |
| In August, | " | 15 | do | do | 9·388 | " |
| In September, | " | 6 | do | do | 2·075 | " |
| In October, | " | 5 | do | do | 2·230 | " |
| In November, | " | 4 | do | do | 7·265 | " |
| In December, | " | 12 | do | do | 11·100 | " |

It is worthy of remark that the quantity of rain that fell in the month of June was fully equal to the average annual quantity for more than twenty years.

